# Evaluation of single and double-locus real-time PCR assays for methicillin-resistant *Staphylococcus aureus *(MRSA) surveillance

**DOI:** 10.1186/1756-0500-3-110

**Published:** 2010-04-21

**Authors:** Sara Kolman, Haya Arielly, Yossi Paitan

**Affiliations:** 1 Clinical Microbiology Laboratory, Meir Medical Center, Kfar Saba, Israel

## Abstract

**Background:**

Methicillin-resistant *Staphylococcus aureus *(MRSA) is a human pathogen, representing an infection control challenge. Conventional MRSA screening takes up to three days, therefore development of rapid detection is essential. Real time-PCR (rt-PCR) is the fastest method fulfilling this task. All currently published or commercially available rt-PCR MRSA assays relay on single or double-locus detection. Double-locus assays are based on simultaneous detection of *mecA *gene and a *S. aureus*-specific gene. Such assays cannot be applied on clinical samples, which often contain both coagulase-negative staphylococci (CoNS) and *S. aureus*, either of which can carry *mecA*. Single-locus assays are based on detection of the staphylococcal cassette chromosome *mec *(SCC*mec*) element and the *S. aureus*-specific *orfX *gene, assuming that it is equivalent to *mecA *detection.

**Findings:**

Parallel evaluation of several published single and double-locus rt-PCR MRSA assays of 150 pure culture strains, followed by analysis of 460 swab-derived clinical samples which included standard identification, susceptibility testing, followed by PCR detection of staphylococcal suspected isolates and in-PCR mixed bacterial populations analysis indicated the following findings.

Pure cultures analysis indicated that one of the single-locus assay had very high prevalence of false positives (Positive predictive value = 77.8%) and was excluded from further analysis. Analysis of 460 swab-derived samples indicated that the second single-locus assay misidentified 16 out of 219 MRSA's and 13 out of 90 methicillin-sensitive *S*. *aureus*'s (MSSA) were misidentified as MRSA's. The double-locus detection assay misidentified 55 out of 90 MSSA's. 46 MSSA containing samples were misidentified as MRSA and 9 as other than *S. aureus *ending with low positive predicted value (<85%) and very low specificity (<62%).

**Conclusion:**

The results indicate that high prevalence of false-positive and false-negative reactions occurs in such assays.

## Introduction

Staphylococci are ubiquitous colonizers of the skin and mucous membranes. Among them, *Staphylococcus aureus *is the most pathogenic species and a leading cause of several life-threatening diseases [1]. *S. aureus *represents a major public health threat, as MRSA strains are the leading cause of nosocomially acquired infections. MRSA infections represent a challenge to both infection control and treatment strategies, resulting in increased morbidity, mortality, length of hospitalization and health care costs [2-4]. Conventional MRSA screening takes up to three days, during which patients and staff carriers can spread MRSA. Development of rapid accurate detection techniques should contribute to the prevention of transmission. rt-PCR is the fastest method fulfilling this task.

All currently published or commercially available rt-PCR MRSA detection assays rely on single or double-locus detection. Double-locus assays are based on simultaneous detection of the *mecA *gene and a *S. aureus*-specific gene, such as *Sa442 *[5]. However, they cannot differentiate between MRSA and a mixture of methicillin-sensitive *S. aureus *(MSSA) and methicillin-resistant coagulase-negative staphylococci (MR-CoNS). As more than 80% of nasal swabs contain staphylococci, and 70-80% of CoNS are MR-CoNS [1,6], many MSSA-containing samples result in a pseudo-MRSA genotype ending with a low positive predictive value (PPV) [6]. Single-locus assays are based on detection of the right extremity of the staphylococcal cassette chromosome *mec *(SCC*mec*) element assuming this is equivalent to *mecA *detection [7,8]. These assays include the first commercial test for MRSA detection that has been approved by the U.S. Food and Drug Administration [8].

As a pre-step for the introduction of direct swab MRSA molecular detection analysis to our culture based MRSA surveillance [9], we assessed the use of single and double-locus rt-PCR, based on previously described assays [5,7,8]. Here we report the occurrence of false-negative and false-positive results in two single-locus SCC*mec:orfX *assays [7,8] and a double locus *mecA-Sa442 *assay [5].

## Methods

### Bacterial strains and cultures

The reference strains used for this study were ATCC33591 (MRSA), ATCC25923 (MSSA), and ATCC12228 (methicillin-susceptible *S. epidermidis *[MSSE]). Other staphylococci strains were all clinical isolates.

### Specimen collection and processing

Clinical swab samples were collected from patients according to a previously defined MRSA infection control strategy [9] using Amies agar gel swabs (Copan Italia S.p.a, Brescia, Italy). Swabs were plated on agar plates (CNA or ChromAgar MRSA [HyLab Ltd., Rehovot, Israel]), followed by DNA extraction from suspect staphylococci colonies for rt-PCR and identification by conventional culture- techniques [10].

### Standard identification and phenotypic susceptibility testing of staphylococcal isolates

Staphylococcal isolates were identified morphologically and biochemically by standard laboratory procedures [10] discriminating MRSA, MSSA, MR-CoNS, and MS-CoNS. Screening for oxacillin resistance and other antibiotic resistant phenotypes was done following CLSI guidelines.

### Bacterial lysis and DNA extraction

Template DNA for rt-PCR was extracted using a fast crude DNA extraction method as previously described [11]. DNA was extracted from 2-4 overnight colonies. Aliquots (4-μl) of the extracted DNA were then transferred to the rt-PCR reaction. No PCR inhibition was observed.

### rt-PCR assays, primers, and probes

Primers and probes for each assay were described previously by Reischl [5] Cuny [7] and Huletsky [8]. As Cuny's assay is a regular PCR assay we used Huletsky's [8] probe with Cuny's primers. Primers and a probe targeting the human β-globin gene were added to each assay as an internal control, validating the absence of PCR inhibition.

Validation assays, screening assays and specificity analyses, were performed in a total volume of 25- μl comprised from 4-μl of crude DNA extract, transferred directly to a 21-μl PCR mixture containing the primers and probes, 12.5-μl of ABsolute™ QPCR (ABgene, Epsom, UK) and 0.15-ng human chromosomal DNA (positive at approximately C_t _of 30) using RG-3000 PCR instrument (Corbett Research, Australia). All runs contained MRSA control (ATCC33591), MSSA control (ATCC25923), MR-CoNS control (clinical isolate MRSE) and two NTC controls. The cycling protocol was: 15 min at 95°C, followed by 40 cycles of 15 s at 95°C, 30 s at 56°C and 15 s at 72°C.

### Validation and specificity of the rt-PCR assay

Validation and specificity of the rt-PCR assays were performed in triplicate, using the reference strains ATCC33591 (MRSA), ATCC25923 (MSSA), and ATCC12228 (MSSE), and 49 clinical MRSA isolates (10 of each type I-IV SCC*mec *and 9 type V SCC*mec *isolates), 49 MSSA isolates, 25 MR-CoNS isolates and 24 MS-CoNS isolates.

### Validation of clinical MRSA screening rt-PCR assays

All isolates were analyzed for the presence of in PCR mixed bacterial populations. Validation of "unusual" genotypic strains was performed by a second identical rt-PCR assay, by agarose gel electrophoresis and by conventional identification. The "unusual" genotypic strains include:*Sa442 *negative MSSA, SCC:*orfX *positive MSSA, oxacillin-susceptible MRSA (*mecA *positive), SCC*mec:orfX *negative MRSA and oxacillin-resistant MSSA (*mecA *negative).

## Results

The validation results of the 150 pure culture strains is presented in Table 1, all internal controls and run controls were identified correctly. As seen in Table 1, one out of 50 MRSA isolate was misidentified by both Cuny's [7] and Huletsky's [8] single-locus detection assays. Two out of 50 MSSA strains were misidentified by both Huletsky's and Reischl's assays, and 14 out of the 50 MSSA were misidentified as MRSAs by Cuny's assay. The results emphasized the high percentage (>25%, PPV = 78%) of false positives using Cuny's [7] assay (Table 1). Consequently, we decided to continue and assess only the Huletsky's and Reischl's assays with clinical samples.

**Table 1:** Validation of pure cultures by single and double-locus detection assays

	Real Time PCR results							
	
Pure culture strains Total n = 150	**Cuny's **[7] single-locus detection^a^		**Huletsky's **[8] single-locus detection^b^		**Reischl's **[5] double-locus detection^c^			
			
	*SCC:orfX+* (MRSA)	*SCC:orfX-* (other)	*SCC:orfX+* (MRSA)	*SCC:orfX-* (other)	*Sa442+ mec+* (MRSA)	*Sa442+ mec-* (MSSA)	*Sa442- mec+* (Oxa. resistant)	*Sa442- mec-* (other)
MRSA (n = 50)	49	1	49	1	50	---	---	---
MSSA (n = 50)	14	36	2	48	---	48	---	2
MR-CoNS (n = 25)	---	25	---	25	---	---	25	---
MS-CoNS (n = 25)	---	25	---	25	---	---	---	25

^a ^sensitivity = 98%, specificity = 86%, Positive predictive value = 77.8%, Negative predicted value = 98.9%

^b ^sensitivity = 98%, specificity = 98%, Positive predictive value = 96.1%, Negative predicted value = 99%

^c ^sensitivity = 100%, specificity = 100%, Positive predictive value = 100%, Negative predicted value = 100%

During a six-month period, we applied both Huletsky's and Reischl's assays to 460 suspect staphylococci colonies, recovered from 4097 swabs, in 60 rt-PCR runs. All samples were also analyzed by conventional methods. The results are presented in Table 2.

**Table 2:** Clinical samples results of single and double-locus detection assays

	Real Time PCR results							
	
Conventional culture identification (n = 338)	**Huletsky's **[8] single-locus detection^a^		**Reischl's **[5] double-locus detection^b^				Combined results^c^	
			
	*SCC:orfX+* (MRSA)	*SCC:orfX-* (other)	*Sa442+ mec+* (MRSA)	*Sa442+ mec-* (MSSA)	*Sa442- mec+* (non S.A oxaR)	*Sa442- mec-* (other)	MRSA^d^	Not MRSA^e^
MRSA (n = 219)	203	16	218	1	---	---	202	17^f^
MSSA (n = 90)	13	77	(44+2)^g^	35	---	9	1^h^	89^i^
MR-CoNS (n = 16)	---	16	---	---	16	---		16
MS-CoNS (n = 8)	---	8	---	---	---	8		8
non-Staph. (n = 5)	---	5				5		5

^a ^sensitivity = 92.7%, specificity = 89.1%, Positive predictive value = 93.4%, Negative predicted value = 86.9%,

^b ^sensitivity = 99.5%, specificity = 61.3%, Positive predictive value = 82.6%, Negative predicted value = 98.6%

^c ^sensitivity = 92.2%, specificity = 99.2%, Positive predictive value = 99.5%, Negative predicted value = 87.4%

^d ^MRSA = *SCC:orfX *positive, *Sa442 *positive, *mec *positive

^e ^Not MRSA = all other results.

^f ^16 *SCC:orfX *negative, *Sa442 *positive, *mec *positive and 1 oxacillin-resistant MSSA (*SCC:orfX *positive, *Sa442 *positive *mec *negative) classified as MRSA by culture methods.

^g ^44 were MSSA mixed with MR-CoNS, 2 were oxacillin-susceptible MRSA classified as MSSA by culture methods.

^h ^1 *SCC:orfX *positive, *Sa442 *positive, *mec *positive originated from a mixture of *SCC:orfX *positive, *Sa442 *positive, *mec *negative MSSA and *mec *positive MR-CoNS

^i ^9 *SCC:orfX *negative, *Sa442 *negative, *mec *negative; 2 *SCC:orfX *negative, *Sa442 *positive, *mec *positive; 22 *SCC:orfX *negative, *Sa442 *positive, *mec *negative; 13 *SCC:orfX *positive, *Sa442 *positive, *mec *negative MSSA's and 43 *SCC:orfX *negative, *Sa442 *positive, *mec*

After exclusion of 122 duplicate patients, out of 338 suspect staphylococci colonies analyzed by conventional identification methods, 219 contained MRSA isolates and 119 were non-MRSA containing isolates. Of these 119 samples, 90 contained MSSA, 16 contained MR-CoNS, 8 contained MS-CoNS and 5 contained non-staphylococcal isolates.

16 and 1 out of 219 MRSAs were misidentified as non-MRSA by Huletsky's assay and by Reischl's assay, respectively. 13 and 46 out of 119 non-MRSA containing samples were misidentified as MRSA (false positives) by Huletsky's assay and by Reischl's assay, respectively. The diagnostic values of the assays are noted in Table 2. A detailed description of all the strains, their genotype and origin is given in the footnotes of Table 2.

The results of the mixed bacterial populations analysis indicated, that of the 219 MRSA containing samples, 37% contained also other Gram-positive bacteria, 6% Gram-negative bacteria and 32% both Gram-positive and Gram-negative. Of the 90 samples containing MSSA, 42% also contained other Gram-positive bacteria, 2% Gram-negative bacteria, and 21% both Gram-positive and Gram-negative. In addition, MR-CoNS were found in 49% of the MSSA-containing samples. It should be noted that those percentages do not represent actual percentage of nasal swabs with mixed populations as many samples originated from oxacillin containing plates.

## Discussion

The major disadvantage of culture based MRSA detection methods is time constraints which can take 2 to 4 days, during which time carriers can spread MRSA. The introduction of rapid molecular MRSA detection should contribute to the prevention of transmission and improved infection control. The aim of this study was to evaluate several published rt-PCR assays for MRSA detection, before introducing direct swab molecular detection to our MRSA surveillance program [9]. To eliminate difficulties associated with direct swab analysis (PCR inhibition, swab extraction protocols, analytical sensitivities, limits of detection, and logistical difficulties of swab collection and transport to the laboratory) swab samples were plated and rt-PCR was done the next day on *S. aureus *suspected colonies.

The main disadvantage of double-locus assays is their inability to differentiate between MRSA and a mixture containing both MSSA and MR-CoNS, resulting in a pseudo-MRSA genotype. Cuny's [7] assay is based on one forward primer of which at least five of its 3' nucleotides are within the *S. aureus orfX *gene (present in MSSA) and an *S. aureus orfX *specific reverse primer. Huletsky's [8] assay is based on a set of five different forward SCC*mec*-specific primers, a *S. aureus orfX*-specific reverse primer and a set of three *S. aureus orfX*-specific probes. The Huletsky assay was the basis for the development of the first commercial test which has been approved by the U.S. Food and Drug Administration for MRSA detection [8].

In contrast to the high accuracy observed by Huletsky's and Reischl's assays when applied on pure cultures, we observed a high percentage of false positives (specificity = 86 and PPV = 77.8%) when applying Cuny's [7] assay with MSSA strains (Table 1). This is probably due to the design of Cuny's forward primer, which overlaps five of its 3' nucleotides with *S. aureus orfX *gene (present in MSSA). The high false positive rate for MSSA's (>25%) reduces the likelihood of its use.

We further assessed Huletsky's and Reischl's assays using clinical samples. A detailed description of all the strains, their rt-PCR assigned genotype and origin is presented in Table 2 and its footnotes. As seen in Tables 1 and 2, we isolated several "unusual" genotypic strains, including 11 *Sa442 *negative MSSAs, 15 SCC:*orfX *positive MSSAs, 2 oxacillin-susceptible MRSA (*mecA *positive, *Sa442 *positive), 17 SCC*mec:orfX *negative MRSAs and 1 oxacillin-resistant MSSA (*mecA *negative, *Sa442 *positive). Work is in progress to further characterize these strains.

In our point of view, the diagnostic values of both assays were unsatisfactory for clinical samples. Reischl's assay displayed low specificity and low positive predictive value, resulting primarily from pseudo-MRSA mixed populations. Huletsky's assay displayed unsatisfactory specificity and unsatisfactory negative predictive value due to the prevalence of SCC*mec:orfX *negative MRSAs and SCC*:orfX *positive MSSAs. The ability to detect these strains in an infection control program is crucial. If carriers of SCC*mec:orfX *negative MRSA's strains are undetected, this strain will spread in the hospital affecting both the infection control program and the clinical outcome. The inability to discriminate SCC:*orfX *positive MSSAs from MRSA and MSSA is clinically less critical. However, the SCC:*orfX *positive MSSA strain can spread in the hospital and increasingly affect the whole infection control program. In addition, based on previous reports, much lower diagnostic are expected for both assays with direct swab analysis [6,12,13].

Users of "single-locus" and "double-locus" MRSA detection assays, both in-house or commercial, applied on samples containing mixed bacterial populations such as direct swab analysis, should be aware of the high prevalence of false-positive and false-negative reactions, specifically in hospitals where it is highly likely that a substantial proportion of the MRSA strains may be clonally related.

## Competing interests

The authors declare that they have no competing interests.

## Authors' contributions

YP carried all the design, coordination, molecular studies, analysis of results and draft the manuscript. SK and HA participated in the standard identification, susceptibility testing of samples and participated in the analysis of results. All authors read and approved the final manuscript.
